# Genetic evaluation of CRISPR-Cas9 off-target effects from deleterious mutations on *Drosophila* male single X chromosome

**DOI:** 10.1007/s10142-025-01775-y

**Published:** 2025-12-10

**Authors:** Wei Bian, David W. J. Mcquarrie, Irmgard U. Haussmann, Roland Arnold, Matthias Soller

**Affiliations:** 1 Division of Molecular and Cellular Function, School of Biological Sciences, University of Manchester, Oxford Road, Manchester, M13 9PT United Kingdom; 2 School of Biosciences, College of Life and Environmental Sciences, University of Birmingham, Edgbaston, Birmingham, B15 2TT United Kingdom; 3 College of Life Science, Birmingham City University, Birmingham, B5 3TN United Kingdom; 4 Department of Cancer and Genomics Sciences, School of Medical Sciences, College of Medicine and Health, University of Birmingham, Edgbaston, Birmingham, B15 2TT United Kingdom

**Keywords:** CRISPR, Cas9, SgRNA, Off-target effect, *Drosophila*

## Abstract

Clustered regularly interspaced short palindromic repeats (CRISPR)-associated nuclease protein 9 (Cas9) is a powerful tool used for genome engineering, but concerns remain about off-target effects. Here we evaluate potential deleterious effects of CRISPR-Cas9 by combining sequence analysis and the genetics of the male X chromosome in a *Drosophila* model. Since males have only one X chromosome deleterious mutations on the X chromosome will manifest in reducing viability or result in visible phenotypes and thus provide sensitive readouts of off-target activity. Our data do not support large scale off-target effects in *Drosophila*. To optimize sgRNA selection, we incorporated off-target evaluation into the PlatinumCRISPr sgRNA selection tool for a broad range of organisms.

**Supplementary Information:**

The online version contains supplementary material available at 10.1007/s10142-025-01775-y.

## Background

A major advance in genome engineering came from the discovery of *Streptococcus pyogenes* clustered regularly interspaced short palindromic repeats (CRISPR)-associated nuclease protein 9 (Cas9) that can induce RNA guided site-specific double-strand breaks in DNA to facilitate homologous recombination by a template provided (Doudna 2020, Garcia-Doval and Jinek 2017, Hille et al. 2018, Jiang and Doudna 2017, Pacesa et al. 2024, Villiger et al. 2024). CRISPR-Cas9-mediated genome editing has been adopted in various research models, including *Drosophila*, and has been extensively studied and refined (Zirin et al. 2022). However, concerns persist regarding possible off-target scission of the Cas9/RNA complex (Cradick et al. 2013, Fu et al. 2013, Hsu et al. 2013, Wang et al. 2016), similar to the well-documented off-target effects of small RNAs used in RNA interference (Chen et al. 2021, Pei and Tuschl 2006, Sudbery et al. 2010). Detailed analysis of Cas9/sgRNA activity revealed variable on-target indel efficiencies ranging from 2.5–50% in different cell lines, and worryingly, off-target indel activity of up to 63% tolerating as many as five mismatches (Fu, et al. 2013, Hsu, et al. 2013, Lin et al. 2014, Wang, et al. 2016). In a large-scale analysis from 79 studies, an average of 5 off-targets per sgRNA were detected from manual curation and validation in cultured cells (1844 for 368 sgRNAs) (Wang et al. 2025). Considerable off-target effects have also been observed in *C. elegans*, zebrafish and mice (Anderson et al. 2018, Hoijer et al. 2022, Medley et al. 2022), but this topic has not thoroughly been addressed in *Drosophila*. Several online tools have been developed to predict potential off targets, these predictions, however, remain suboptimal, illustrating the need for improvement (Guo et al. 2023). This need has gained further importance with the US FDA approval of the first CRISPR-based gene therapy in 2023 (Mullard 2024).

In *Drosophila*, the X chromosome accounts for 20% of the *Drosophila* genome and contains around 820 lethal genes (Peter et al. 2002), making it a strong indicator for detecting deleterious off-target effects induced by CRISPR-Cas9/sgRNA DNA scission complex. Here, we use male viability and visual phenotypes as sensitive genetic readouts for potential deleterious X-linked off-target mutations, since males possess only one X chromosome. Accordingly, deleterious mutations will manifest in lethality or visible phenotypes, because no other copy is present as is the case for two copies of autosomes. We include four *nosCas9* insertion lines (*nosCas9*^*2-w+*^, *nosCas9*^*3-w+*^, *nosCas9*^*2-GFP*^ and *nosCas9*^*3-GFP*^) and 5 pairs of sgRNAs each targeting non-essential autosomal genes (*Ythdf, Ythdc1, Oatp58Da-c, Nsun6* and *RBM5*). Our analysis revealed no significant reduction in male viability or display of visible phenotypes, suggesting that CRISPR-Cas9 does not induce widespread deleterious X-linked off-target mutations in *Drosophila*. We note, however, that this approach primarily detects lethal or visible mutations on the X chromosome and does not assess neutral or autosomal off-targets, which are discussed as limitations. We further integrated our off-target evaluation metrics into the PlatinumCRISPr sgRNA design platform (https://platinum-crispr.bham.ac.uk) and provide an improved framework for selecting highly specific sgRNA across diverse organisms.

## Results and discussion

**Fig. 1:**
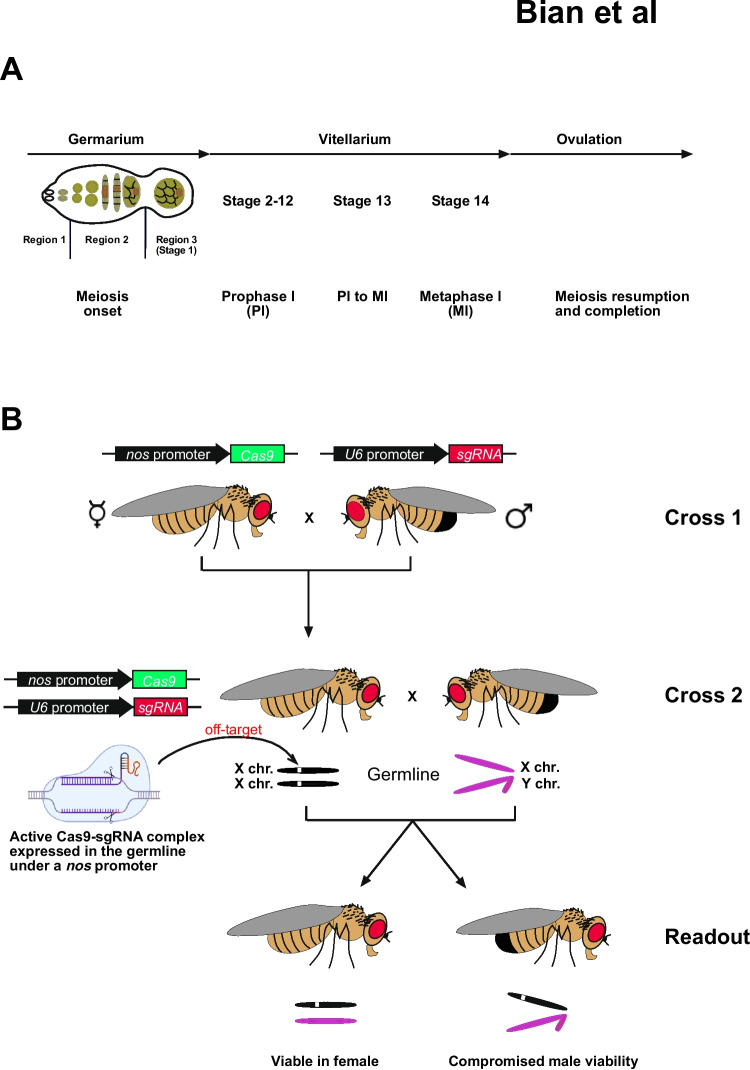
Schematic of *Drosophila* germline differentiation and crossing scheme. **A**) Schematic depiction of the *Drosophila* germline and oogenesis with respect to the occurrence of meiosis. **B**) Crossing scheme to test off target effects on the X-chromosome of sgRNAs targeting non-vital autosomal genes. Off-target mutations can occur in the germline of females expressing both Cas9 and the sgRNA forming an active genome editing complex (Cross 2). To measure such off-target effects male viability and visual phenotypes are scored as readouts in the progeny of Cross 2 relative to females

Here we used *Drosophila* to assess whether the Cas9-sgRNA complex would induce deleterious off-target effects on the single copy X chromosome in males. For CRISPR-Cas9 genome editing in *Drosophila*, Cas9 is generally expressed by a germline promoter (e.g. *nanos* or *vasa* promoter) and sgRNA is ubiquitously expressed under a U6 snRNA promoter. Within the germline, starting from the onset of meiosis in the germarium to completion in the egg just before fertilization and laying, CRISPR-Cas9 genome editing can take place at any stage driven by the germline promoter (Figure 1A) (Gavis et al. 1996a, Gavis et al. 1996b, Kondo and Ueda 2013, Ren et al. 2013, Van Doren et al. 1998, Wang and Lehmann 1991, Wang and Lin 2004). To assess sgRNAs for off-target effects, *nosCas9* females were crossed with males ubiquitously expressing a pair of sgRNAs under the control of a U6a and U6c promoter flanking the target gene distal (sgRNA1) and proximal (sgRNA2) to generate a complete gene deletion (Cross 1, Figure 1B). The progeny from this cross now expresses both Cas9 and sgRNA, forming a genome-editing-competent complex in their germline. These females were then outcrossed to wild-type males (Cross 2, Figure 1B). If off-target mutations occur in essential genes on the X chromosome, male viability will be compromised in the progeny of Cross 2 (Readout, Figure 1B). Hence, the F1 generation of offspring from Cross 2 were scored according to sex and visual phenotypes upon eclosion, and male viability was determined as the ratio of surviving males to females.

**Fig. 2:**
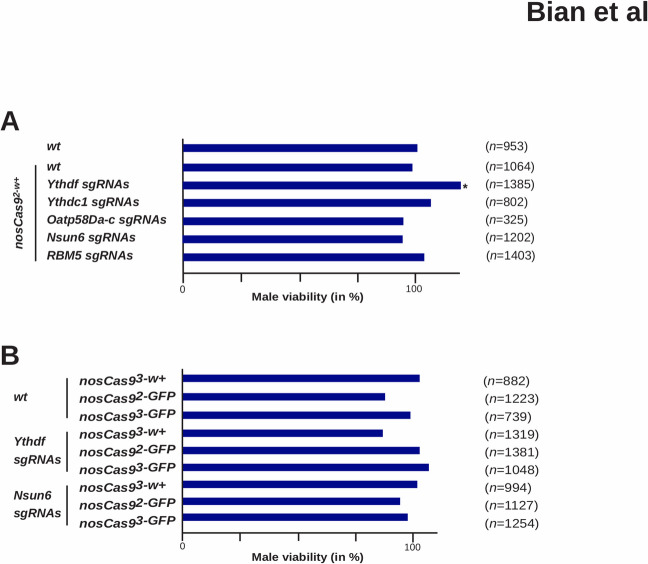
CRISPR-Cas9 induces no large-scale deleterious off-target effects. **A**) Viability of males in wild type, *nosCas9*^*2-w+*^ and *nosCas9*^*2-w+*^ in the presence of either *Ythdf, Ythdc1, Oatp58Da-c, Nsun6* and *RBM5* sgRNAs is shown as percentage of the number of female flies from the same cross. Statistically significant differences are indicated by an asterisk (* *p*≤0.05). **B**) Viability of males in the presence of *nosCas9*^*3-w+*^, *nosCas9*^*2-GFP*^ and *nosCas9*^*3-GFP*^ in the absence and presence of either *Ythdf* or *Nsun6* sgRNAs is shown as percentage of the number of female flies from the same cross.

We tested 10 different sgRNAs to make complete gene deletions for five targets (*Ythdf, Ythdc1, Nsun6,* *RBM5* and paralogues *Oatp58Da-c)* using one sgRNA in the beginning and one sgRNA at the end of the gene or in case of *Oatp58Da-c before OATP58Da* and after *OATP58Dc* to delete all three paralogues (Fig. 2 and Supplemental Table 1)*.* All target genes are located on either second or third chromosome, where we had generated deletions for these genes and validated that null mutants are viable. For these experiments, Cas9 was expressed only in the germline using a *nos* promoter and we tested 4 different *nosCas9* insertion lines (*nosCas9*^*2-w+*^, *nosCas9*^*3-w+*^, *nosCas9*^*2-GFP*^ and *nosCas9*^*3-GFP*^). To set up the crosses, *nosCas9* females and males expressing a pair of sgRNAs were used because the *nos* promoter exhibits substantially higher transcription activity in adult females than in males (FlyBase 2025, Wang and Lehmann 1991). As control groups, male viability was determined in a cross of the wildtype strain and in a cross of *nosCas9* females to wildtype males (Figure 2A and B**)**.

We compared male viability and visual phenotypes as readouts for single X chromosome aberrations to the heteroallelic combination of two X chromosomes in females. Reduced viability or visible phenotypes were not observed for F0 individuals nor for the control groups (Figure 2A). For none of the five double sgRNA combinations and four *nosCas9* inserts we found that male viability was reduced (Fig. 2). For *Ythdf* sgRNAs, male viability was marginally increased (*p*=0.04) with *nosCas9*^*2-w+*^, but not the other three Cas9 inserts (Figure 2A, Supplemental Table 1). Since YTHDF is a reader for *N*^6^-methyladenosine (m^6^A) containing RNA and is involved in the regulation of *Sxl* in *Drosophila* sex determination and dosage compensation, it is possible that female viability was reduced due to *Ythdf* removal rather than male viability being enhanced (Bawankar et al. 2021, Haussmann et al. 2016). Despite our focus on deleterious X-linked off-target mutations, it is important to note that off-targets in autosomes or neutral mutations might occur, but detection of such mutations would require either isogenic strains and large scale deep-genome sequencing (1000x or more) to distinguish true de novo mutations from polymorphisms in the populations (Mackay et al. 2012).

We then evaluated whether any of the sgRNAs had matches in the coding regions of lethal genes on the X chromosome. Off-target candidates were computed for the 19 nucleotides before the PAM site allowing up to five mismatches in the absence or presence of one bulge nucleotide (Fu, et al. 2013, Lin, et al. 2014) using CAS-OFFinder (Bae et al. 2014) for the *Drosophila* genome (Dmel R65.6). Phenotype information was obtained from FlyBase under downloads and filtered for lethal genes. Using these criteria, we identified 764 target sites in 182 lethal genes on the X chromosome potentially resulting in lethality if hit (Supplemental Table 1).

Taken together, our results reveal no significant off-target damage to the single copy X chromosome by the CRISPR-Cas9 DNA scission complex by un-targeted activity. To assess the potential impact of off-target activity, we considered previous studies in culture cells. Here, Fu et al. (2013) detected off-target effects for 4 out of 6 sgRNAs, with an average of 18.6±6.3% off-target indels compared to 41.9±11.4% on target indels in U2OS.EGFP cells. If comparable off-target activity had occurred in our experiments, we would have expected to observe a reduction in male viability. Also, single-nucleotide scanning mutagenesis at all 20 positions of an sgRNA targeting the *white* locus in *Drosophila* did not result in detection of white-eyed flies, which is consistent with low off-target activity (Ren, et al. 2014). In mice, CRISPR-induced mutagenesis can induce off-target effects, but seems generally to be rare (Aryal et al. 2018), and others have not found off-target effects at all (Iyer et al. 2018, Luo et al. 2019). In zebrafish, some sgRNAs can produce considerable off-target hits; notably, four such sgRNAs were not predicted by the PlatinumCRISPr tool (Hoijer, et al. 2022), but under which circumstances incorrect sgRNA folding contributes to off-target hits needs to be evaluated.

Imprecise transposon excision was used before CRISPR-Cas9 genome editing to generate *Drosophila* gene knock-outs, but high mutation efficiency was often observed in the absence of a repair template (Haussmann, et al. 2016, Haussmann et al. 2022, Soller et al. 2006). Potentially, the high incidence of off-target effects of Cas9 in cell culture experiments could be attributed to a compromised DNA damage repair system inherent to cancer-derived cell culture cells (Karran and Bignami 1994). Although the Cas9-sgRNA complex is active through-out the germline, it might be most efficient after completion of meiosis II, when no repair template is present.

Moreover, CRISPR-Cas9 DNA scission activity is highly specific, but chronic exposure to the CRISPR-Cas9 DNA scission complex as used in cultured cells might be a source for off-target chromosome aberrations, which can be alleviated by an inducible Cas9 gene (Zirin et al. 2022). Potentially, the higher GC content of human genes compared to *Drosophila* could lead to higher off-target effects if sgRNAs are chosen with a high GC content.

In any case, we have updated the PlatinumCRISPr tool to display off-targets with up to five mismatches (Haussmann, et al. 2024). Since only the 15 nucleotides before the PAM site are strictly required for Cas9 DNA scission (Haussmann, et al. 2024), a higher incident rate for mismatches is apparent in the first four nucleotides (Fu, et al. 2013).

## Conclusions

Our study using *Drosophila melanogaster* as a genetic model demonstrates that sgRNA-guided CRISPR-Cas9 activity did not lead to genetically detectable deleterious off-target effects on the single-copy X chromosome. Despite extensive assessment across multiple *nosCas9* and sgRNA lines, we did not observe consistent reductions in male survival or visual phenotypes, indicating minimal off-target mutagenesis *in vivo*. These results suggest that off-target cleavage events by the Cas9/sgRNA complex are negligible under physiological conditions *in vivo*, contrasting with higher off-target rates observed in cultured cell (Cradick et al. 2013, Fu et al. 2013, Hsu et al. 2013).

By incorporating our off-target evaluation metrics into the PlatinumCRISPr sgRNA design platform (https://platinum-crispr.bham.ac.uk), we provide an improved framework for selecting highly specific sgRNA based on correct sgRNA folding across diverse organisms. Together, these results reinforce the precision of CRISPR-Cas9 genome editing *in vivo* and contribute to the development of more robust tools, enhancing the reliability of CRISPR-based genome engineering.

## Methods

### Drosophila genetics and statistics

A *Drosophila melanogaster yw* strain was used as a wild-type control. Flies were reared at 25° C in plastic vials on standard cornmeal/yeast-rich medium (1% agar, 2% yeast, 7% dextrose, 8% cornmeal w/v and 2% Nipagin from a 10% solution in ethanol) with a 12:12 hour light-dark cycle. The second and third chromosome *nosCas9* fly lines used in this study were either marked with *w+* (Bloomington #78781 and #78782) or *paxGFP* (FlyORF) and were inserted at *attP40* or *attP2* sites, respectively. To select efficiently cutting sgRNAs, the PlatinumCRISPr tool was used (Haussmann, et al. 2024). For each target, two sgRNAs flanking the target region were used to generate complete gene deletions. sgRNA sequences and target regions have been summarized in Supplementary Table 1. Note that deleted fragments can reinsert and rescue mutant phenotypes, hence we have now changed our approach to make knock-outs by generating partial gene deletions (Haussmann, et al. 2024 and knock-out guide on PlatinumCRISPr). All sgRNAs were cloned into *pUC 3GLA* to be expressed under *U6a* and *U6c* promoters as described (Haussmann, et al. 2024). Insertion of sgRNA constructs was achieved as follows: *Ythdc1* sgRNAs were inserted at the *attP40* site, *Ythdf* and *Nsun6* sgRNAs were inserted at the *76A* site, and *Oatp58Da-c* and *RBM5* (*CG4887* and *CG4896*) sgRNAs were inserted at the *attP2* site. F1 male viability was calculated as the percentage of the number of females of the same cross. Significance was calculated by the *Chi*-squared statistical test with FDR corrected significance value of *p*<0.05 (GraphPad Prism).

### Sequence analysis

To identify the coding regions of essential genes on the X chromosome, phenotype information was obtained from Flybase under downloads (3.5.5.5 phenotypic data, "genotype_phenotype_data_fb_2024_06.tsv”) and filtered for lethal genes. Off-target candidates were computed for all guide RNAs (19 bp) using CAS-OFFinder (Bae, et al. 2014) for the *Drosophila* genome (Dmel R65.6) allowing for up to 5 mismatches and bulge size 1 in either sgRNA or target DNA. DNA scission sites were limited to exons by aligning the PAM site to the 5` exon and exceeding 6 nucleotides from the 5` splice site at the other end of the exon.

## Supplementary Information

Below is the link to the electronic supplementary material.

Supplementary file1 (XLSX 62 KB)

## Data Availability

All data generated or analysed during this study are included in the supplementary information files.
